# Spatial Navigation Impairment Is Associated with Alterations in Subcortical Intrinsic Activity in Mild Cognitive Impairment: A Resting-State fMRI Study

**DOI:** 10.1155/2017/6364314

**Published:** 2017-09-20

**Authors:** Zhao Qing, Weiping Li, Zuzana Nedelska, Wenbo Wu, Fangfang Wang, Renyuan Liu, Hui Zhao, Weibo Chen, Queenie Chan, Bin Zhu, Yun Xu, Jakub Hort, Bing Zhang

**Affiliations:** ^1^Department of Radiology, Affiliated Drum Tower Hospital of Nanjing University Medical School, Nanjing, China; ^2^The Czech Brain Aging Study, Memory Clinic, Department of Neurology, Charles University 2nd Faculty of Medicine and Motol University Hospital, Prague, Czech Republic; ^3^International Clinical Research Center, St. Anne's University Hospital Brno, Brno, Czech Republic; ^4^Department of Neurology, Affiliated Drum Tower Hospital of Nanjing University Medical School, Nanjing, China; ^5^Philips Healthcare, Shanghai, China; ^6^Philips Healthcare, Shatin, Hong Kong

## Abstract

Impairment of spatial navigation (SN) skills is one of the features of the Alzheimer's disease (AD) already at the stage of mild cognitive impairment (MCI). We used a computer-based battery of spatial navigation tests to measure the SN performance in 22 MCI patients as well as 21 normal controls (NC). In order to evaluate intrinsic activity in the subcortical regions that may play a role in SN, we measured ALFF, fALFF, and ReHo derived within 14 subcortical regions. We observed reductions of intrinsic activity in MCI patients. We also demonstrated that the MCI versus NC group difference can modulate activity-behavior relationship, that is, the correlation slopes between ReHo and allocentric SN task total errors were significantly different between NC and MCI groups in the right hippocampus (interaction *F* = 4.44, *p* = 0.05), pallidum (*F* = 8.97, *p* = 0.005), and thalamus (*F* = 5.95, *p* = 0.02), which were negative in NC (right hippocampus, *r* = −0.49; right pallidum, *r* = −0.50; right thalamus, *r* = −0.45; all *p* < 0.05) but absent in MCI (right hippocampus, *r* = 0.21; right pallidum, *r* = 0.32; right thalamus *r* = 0.28; all *p* > 0.2). These findings may provide a novel insight of the brain mechanism associated with SN impairment in MCI and indicated a stage specificity of brain-behavior correlation in dementia. This trial is registered with ChiCTR-BRC-17011316.

## 1. Introduction

Deterioration of spatial navigation (SN) skills is often present early in the course of Alzheimer's disease (AD), at least at the stage of mild cognitive impairment (MCI), and has a serious impact on the quality of patients' daily life [1–5]. Therefore, better understanding of the neural mechanisms of SN impairment in AD and MCI might aid the timely diagnosis and intervention in patients within MCI and AD.

Based on an extensive work in animals and humans, two basic strategies of SN have been widely recognized: egocentric (self-based) and allocentric (world-based) [2, 6], and their association with brain regions were investigated [7–9]. The subcortical regions may play a critical role in the SN [3, 6, 10, 11]. The allocentric SN has been mainly associated with the hippocampus, especially the right and posterior hippocampus [3, 6, 8, 11], whereas the striatum and caudate have been involved in egocentric SN processing [3, 6, 12, 13]. Moreover, an altered activation during spatial navigation task within these areas have been found in AD and MCI patients and have been associated with the SN impairment [14].

Intrinsic brain activity consumes over 95% of the brain's energy and is believed to play a critical role in brain function [15, 16]. Therefore, the resting-state functional magnetic resonance imaging (rs-fMRI) may be a useful tool to detect brain dysfunction associated with AD and MCI. To date, alterations in the intrinsic brain activity have been associated with cognitive impairment in both AD and MCI [17–19]. The aberrant intrinsic activities in the subcortical regions in MCI and AD patients have been previously reported [20–22]. However, the studies investigating the relationship between the subcortical intrinsic activity and the SN impairment in AD or MCI are still lacking.

In the current study, our primary objective was to assess whether the subcortical intrinsic activity is associated with the SN impairment in patients with MCI. We hypothesized that the SN impairment would be related to abnormal intrinsic brain activity within subcortical areas.

## 2. Material and Methods

### 2.1. Participants

This study was performed according to the Declaration of Helsinki and approved by the institutional review boards of Nanjing Drum Tower Hospital. Written informed consent was obtained from all participants before they were included. 60 participants in total (67.7 ± 11.2 years; range, 40 to 87 years) were recruited from May 2015 to January 2016, including MCI patients (*n* = 33, 68 ± 13 years old) and NC (*n* = 27, 65 ± 12 years old). All were right-handed and underwent a series of standardized clinical assessments including neuropsychological, neurological, and psychiatric evaluations. Participants would be excluded if they had a positive history of major neurological or psychiatric disorder other than AD/MCI, drug, or alcohol abuse and intracranial findings that might contribute to cognitive impairment (e.g., cortical infarcts, hydrocephalus).

### 2.2. Cognitive Assessment and Diagnosis

Patients with MCI were diagnosed by experienced neurologists, according to Petersen criteria [23]: (1) memory complaints observed by caregiver, (2) objective evidence for memory impairment, (3) relatively preserved general cognition for age, (4) essentially intact activities of daily living, and (5) not diagnosed as having AD according to the NINCDS-ADRDA criteria [24]. The NC was defined as (1) no cognitive complaints, (2) normal level of clinical rating scales, (3) no neurological and psychiatric disease history, and (4) not taking any psychoactive medications. Finally, 33 MCI and 27 NC met these criteria and were included in the study.

#### 2.2.1. Spatial Navigation Assessment

**Figure 1:**
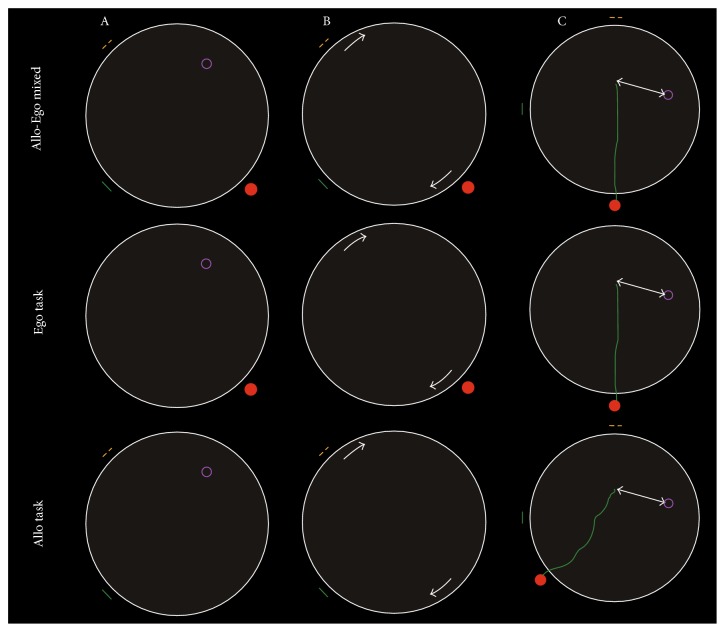
The illustration of the SN tasks. The start point was a red solid circle, the goal was a purple hollow circle, and two orientation points for allocentric reference were a green bar and a set of yellow points. (A) All the points were shown to participate in Allo-Ego mixed task (upper row), start point and goal were shown in Ego task (middle row), and orientation points and goal were shown in Allo task (bottom row). (B) After proper instruction and training trials, the goal is hidden, and then the whole arena rotates with a random angle clockwise or anticlockwise to a new position. (C) The participant is required to draw the route from the start point to the goal on the screen using the mouse. The distance between the participants' chosen goal and correct goal is recorded (the total distance error).

SN accuracy was measured in all participants using a computerized two-dimensional Amunet test battery (NeuroScios, Austria, Gmbh) administered on the computer screen, which used similar spatial navigation paradigm as the hidden goal task published in previous studies [2, 7–9, 25]. Amunet is a modified human analogue of the original Morris water maze (MWM) task, which has been originally designed and used long-term to test both allocentric (world-centered) and egocentric (self-centered) SN strategies in rodents. The human MWM version has been optimized for two-dimensional computer setting and licensed as Amunet tests (Figure 1).

The Amunet test battery has three phases administered in the prescribed order from more simple to more complex. The main objective of all three SN tasks was always to find a goal. This goal was shown at the beginning of the test to the participant and then the goal was hidden: (1) The mixed allocentric-egocentric subtask (Allo-Ego mixed) was the least demanding and was used to get familiarized with the SN tasks. As illustrated in Figure 1, participant was presented with a computer screen that showed a large circle. This circle was an arena with 280 pixels in diameter on a 640 × 480 pixel screen. The participant was asked to find the goal using its spatial relationship with both, the start position (red dot on the arena perimeter) and the two orientation cues on the arena perimeter (yellow and green). In the beginning, the correct goal position was disclosed to the participant along with start and orientation cues to understand mutual spatial relationships. Subsequently, the goal was hidden and the participant was required to locate it and draw the route from start to presumed goal on the screen using the mouse. (2) In egocentric (Ego) task, the participant could only use the start position and its relationship (distance and direction) to hidden goal, whereas the orientation cues were not displayed (Figure 1, middle row). (3) In the allocentric (Allo) task, the participant could only use two orientation cues, whereas the start position he/she starts from was chosen randomly and was unrelated to the goal or the orientation cues (Figure 1, bottom row).

Each task, that is, Allo-Ego mixed, Ego, and Allo, involved 8 trials. SN performance was automatically recorded during the examination. Spatial navigation accuracy was reported as the total distance error (in pixels) between goal position chosen by the participant and the correct goal position across all eight trials of a given task. The SN tasks were not time restricted.

### 2.3. MRI Acquisition

MR images were acquired on two 3T MRI scanners (Philips, Achieva TX and Ingenia, the Netherlands). Both rs-fMRI and T_1_-weighted high-resolution structural MRI acquisitions. T_1_-weighted images were acquired with the following parameters: 192 sagittal slices, repetition time (TR) = 9.74 ms, echo time (TE) = 4.60 ms, slice thickness = 1 mm, field of view (FOV) = 256 × 256 mm^2^, and voxel size = 1.00 × 1.00 × 1.00 mm^3^ on Achieva TX scanner; 222 sagittal slices, repetition time (TR) = 7.65 ms, echo time (TE) = 3.43 ms, slice thickness = 0.8 mm, field of view (FOV) = 256 × 256 mm^2^, and voxel size = 0.8 × 0.762 × 0.762 mm^3^ on Ingenia scanner. rs-fMRI data were obtained using an echo-planar imaging (EPI) sequence with the following parameters: 35 axial slices, TR = 2 s, TE = 30 ms, slice thickness = 4.0 mm, FOV = 192 × 192 mm^2^, and voxel size = 3.0 × 3.0 × 4.0 mm^3^, with 230 volumes on Achieva TX scanner and with 37 axial slices, TR = 2 s, TE = 30 ms, slice thickness = 3.0 mm, FOV = 192 × 192 mm^2^, and voxel size = 1.5 × 1.5 × 3.0 mm^3^, with 230 volumes on Ingenia scanner. During the image acquisition, participants rested in the supine position with their head snugly fixed by foam pads to minimize head movement. During the rs-fMRI acquisition, participants were instructed to close their eyes, to remain still and calm, to not think systematically about anything and to not fall asleep. To control the potential interference from different scanner usage, scanner type was added as a categorical covariate in a linear regression in all further statistical analyses [26].

### 2.4. Resting State Functional and Structural MRI Processing

The rs-fMRI volumes were processed using the Data Processing Assistant for Resting-State fMRI (DPARSF) [27] and the Resting-State fMRI Data Analysis Toolkit (REST) [28], including the following steps: (1) discarding the first 10 volumes; (2) slice timing; (3) head motion correction; and (4) regressing out of nuisance variables (15 in total), including 6 head motion parameters and their derivatives, the average CSF and WM signal, and the linear term. Participants were excluded from our final analysis if their maximum head motion translation was larger than 2 mm or their maximum rotation was larger than 2°. Accordingly, 17 participants in total (11 NC and 6 MCI) were excluded from further analyses due to incomplete acquisition or poor image quality.

The local intrinsic activity within subcortical regions was estimated using three local rs-fMRI measures, including the amplitude of low-frequency fluctuation (ALFF) [29], fractional ALFF (fALFF) [30], and regional homogeneity (ReHo) [31]. All of these measures were calculated from the preprocessed data in the native EPI space individually and all rs-fMRI volumes were preresampled into same voxel size (3 × 3 × 3 mm^3^) to achieve comparability. The ALFF and fALFF values were derived from frequency domain analyses of the fMRI signal within each voxel, as previously reported [29, 30]. The ReHo value within each voxel was defined as the Kendall's coherence coefficient among the time courses of this voxel and its 26 neighbor voxels [31].

FIRST was used to segment the bilateral accumbens, putamen, palladium, hippocampus, caudate, amygdala, and thalamus, in total 14 regions of interest (ROI) based on the T_1_-weighted volume [32]. Then T_1_-weighted volume was registered to the fMRI volume for each participant individually. The mean ALFF, fALFF, and ReHo values within these ROIs were then extracted along with the ROI volumes.

### 2.5. Statistical Analysis

The chi-square test was used to calculate the between-group differences in categorical variables, whereas two sample *t*-tests were used for continuous variables. The ALFF, fALFF, and ReHo values from subcortical ROIs were compared between NC and MCI groups using two sample *t*-tests including age, gender, scanner type, and years of education as covariates. ROI volumes adjusted for the total intracranial volumes were also compared between groups. In further analyses, we only focused on those subcortical regions where an abnormal local ALFF, fALFF, or ReHo were measured. Namely, if an rs-fMRI measure showed significant group differences in one ROI, Pearson's correlations between SN total distance error in Allo, Ego, and Allo-Ego mixed task and such rs-fMRI measure in the corresponding ROI was calculated, for MCI group and then for NC group. Furthermore, we evaluated whether the correlation between SN performance and rs-fMRI measures has a statistical difference between MCI and NC. Specifically, we included all of the MCI and NC participants in one linear model, taking the rs-fMRI measure and SN total distance error as independent and dependent variable, respectively. A *F*-test was utilized to evaluated the effect of “group × total error” interaction term (here, group is a categorical variable marking one participant as MCI or NC). Age, gender, scanner type, and years of education were also adjusted. The significance was set at *p* < 0.05. All these statistical analyses were performed by SurfStat package (http://www.math.mcgill.ca/keith/surfstat/).

## 3. Results

Information on the demographics, clinical evaluations, and SN accuracy in Allo, Ego, and Allo-Ego mixed tasks for MCI and for NC groups were listed in Table 1. The MCI and NC differed in years of education (*t* = 3.48, *p* = 0.001) and gender (*χ*^2^ = 16.19, *p* = 0.001) but not in age (*t* = 1.91, *p* = 0.06) or scanner type (*χ*^2^ = 3.83, *p* > 0.05). The total distance error in Ego task was higher in the MCI compared to the NC group (*t* = −2.12, *p* = 0.04). However, no difference was found in Allo or mixed Allo-Ego SN tasks.

**Figure 2:**
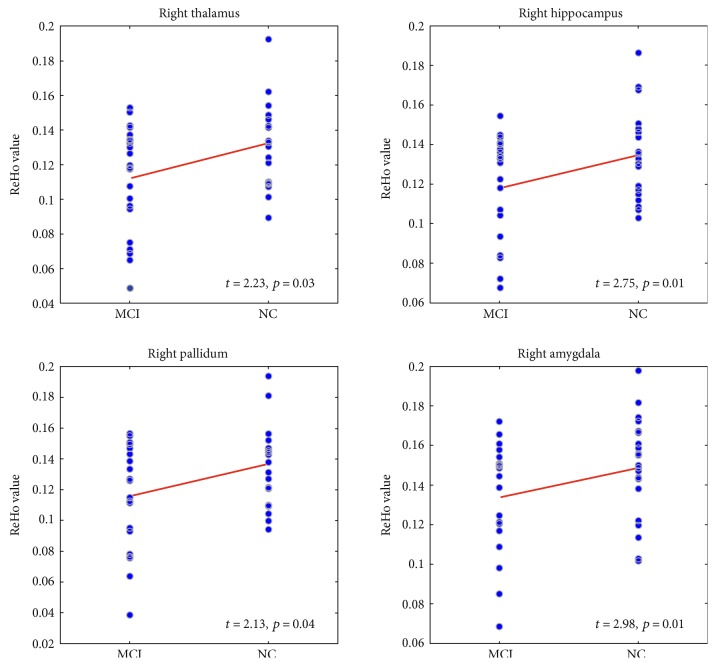
The scatter plots showed the differences in ReHo values within subcortical regions between MCI and NC.

The ALFF, fALFF, and ReHo value along with subcortical ROI volumes were listed in Table 2. After controlling for age, gender, education, and scanner type, only the ReHo was significantly lower in the MCI group in the right thalamus (*t* = 2.24, *p* = 0.03), right hippocampus (*t* = 2.75, *p* = 0.01), right pallidum (*t* = 2.13, *p* = 0.04), and right amygdala (*t* = 2.98, *p* = 0.01) compared to the NC group (Figure 2). The ALFF, fALFF and volumes of other subcortical regions did not differ between groups.

**Figure 3:**
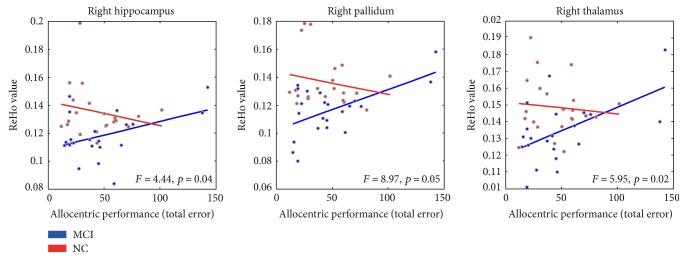
The scatter plots show the differences between clinical groups MCI and NC on allocentric SN performance and ReHo values in the right hippocampus, pallidum, and thalamus. SN accuracy is represented by the total error (distance between subject's guess and correct goal position). In the MCI group, the participants with poorer allocentric spatial navigation skills have higher ReHo values, whereas in the NC group, this is inversed.

The correlations between SN accuracy in Allo, Ego and Allo-Ego mixed tasks and the ReHo values within right thalamus, hippocampus, pallidum and amygdala are shown in Table 3. In the NC group, higher ReHo correlated with smaller total errors in Allo task in the right thalamus (*r* = −0.49, *p* = 0.05) and the right pallidum (*r* = −0.50, *p* = 0.04), and there was a trend in right hippocampus (*r* = −0.45, *p* = 0.07). No correlations between SN performance and rs-fMRI measures were observed in the MCI group (all *p* > 0.2). Furthermore, we found a strong interaction effect that emphasized these correlational results as significant different between MCI and in NC, in right hippocampus (*F* = 4.44, *p* = 0.04), right thalamus (*F* = 5.95, *p* = 0.02) and right pallidum (*F* = 4.28, *p* = 0.05), as illustrated in Figure 3. For Ego or Allo-Ego mixed tasks, there was no significant correlation between ReHo and total distance error in either MCI or NC groups (Table 3).

## 4. Discussion

In the current study, we used various rs- fMRI variables of ALFF, fALFF and ReHo to explore the alterations of subcortical intrinsic activity and their relationship with SN skills in the MCI patients compared to the NC. The ReHo values in right hippocampus, right pallidum, right thalamus and right amygdala were reduced in the MCI compared to the NC group. Moreover, the correlations between higher ReHo values and lower total distance error in Allo task (which means better allocentric spatial navigation performance) in right hippocampus, right pallidum and right thalamus were found in NC but not in MCI. Overall, the intrinsic activity was impaired in subcortical areas in our MCI patients and its association with allocentric SN performance was also disrupted in these patients.

ReHo value was decreased in the right side network of structures comprising the hippocampus, pallidum, amygdala and thalamus [20–22]. Previous studies demonstrated that atrophy of right hippocampus has been associated with allocentric SN impairment in AD and MCI patients [8, 11]. Moreover, the pallidum and thalamus have been involved in self-orientation determination and spatial-related memory in both animal models [33, 34] and humans [10, 13], also an important nuance of SN. In line with these previous studies, the ReHo reduction shown in the current study provided new evidence that the MCI patients also have dis-synchronism of local neural intrinsic activity within the SN networks [31, 35].

Moreover, the lower right thalamic, pallidal and hippocampal ReHo were correlated with greater Allo task total errors in the NC group, which means NC with higher ReHo within these areas has better allocentric spatial navigation skills. This was in line with the previous studies showing important role of these regions in human spatial navigation [8, 11, 14]. However, in the MCI group, these correlations observed in the NC were disrupted, as shown by different correlational tend lines (Figure 3), opposite *r* values in the MCI group compared to the NC group (Table 3) and significant interaction effects (Table 3). These results indicated that the neural degeneration progress within these regions did not only lead to a deficit of ReHo in the MCI compared to the NC, but also disrupted the association between ReHo and Allo SN performance. It was also possible that although there were impairment in these regions as a decreased ReHo reflected, there were some potential compensatory mechanism (noting that Allo task distance error is not severely decreased), and therefore we could not predict the SN task performance by subcortical ReHo in the current mixed MCI sample as well as in NC. However these speculations need further validation in the future. Additionally, note the disruption of the ReHo-SN performance association did not necessarily mean a significant group difference of ReHo, and vice versa. Therefore, these results also highlighted the group specificity should be taken into consider when investigating the association between fMRI measures and human cognitive abilities in dementia studies in the future.

The egocentric SN accuracy was significantly impaired in the MCI group in our study (Table 2). However, we failed to find any association between the subcortical rs-fMRI measures and Ego task performance in the current study. Given that egocentric SN were widely reported to be also related to cortical areas such as the parietal cortex and precuneus [3, 36], whole brain or cortical SN network analysis may be needed with a larger sample size in the future.

We also failed to find any atrophy in subcortical regions and deficit of Allo task performances in the MCI groups, which were reported in previous studies [2, 8, 37]. Possible explanation was that our MCI patients were still mildly impaired and the regional atrophy as well as allocentric SN impairment was not severe enough to be detected. Unfortunately, we were not able to further investigate this without longitudinal sample or pathological validation of patients' diagnosis. On the other hand, our significant results of ReHo may suggest that functional disorder may happen before atrophy during the SN impairment of MCI.

Finally, a number of limitations need to be acknowledged. Firstly, we have relatively a small sample size and the data were collected by two different scanners. Also, education history and gender were different between MCI and NC. Although we always took these factors as covariates in the correlation linear model, they may still have influences on the results. Another limitation is that our computer-based battery of SN tests might not be as sensitive as those in the real environment to spatial navigation skill impairment. Moreover, there is no longitudinal data to confirm if aberrant rs-fMRI measures precede SN impairment and we were also unable to distinguish amnestic MCI from nonamnestic MCI, given that they may have different SN impairment mechanisms [5, 8, 38]. The studies including larger sample and longitudinal data are however needed to confirm our findings.

## 5. Conclusion

The current study demonstrated the association between the subcortical brain intrinsic activity and spatial navigation impairment in NC patients but not in MCI. Subcortical ReHo measure could be a potential predictor of MCI, and revealed the new sights into the neural mechanism of SN impairment.

## Figures and Tables

**Table 1:** Subjects' characteristics; mean (SD).

	NC (*n* = 21)	MCI (*n* = 22)	*t* value/*χ*^2^	*p* value^∗^
MOCA	27.8 ± 2.3	22.0 ± 2.6	7.74	10^−9^
MMSE	29.0 ± 1.0	26.2 ± 2.7	4.46	10^−4^
Allo-Ego navigation error^∗∗^	34.6 ± 16.9	48.7 ± 45.0	−1.35	0.19
Allocentric navigation error^∗∗^	37.0 ± 23.7	51.1 ± 35.0	−0.47	0.64
Egocentric navigation error^∗∗^	44.2 ± 17.2	48.5 ± 40.0	−2.12	0.04
Age (y)	70.7 ± 10.9	64.3 ± 11.1	−1.91	0.06
Education (y)	15.2 ± 2.3	11.6 ± 4.2	−3.48	0.001
Gender (M/F)	7/14	12/10	16.19	0.001
Scanner (T/I)^∗∗∗^	13/8	11/11	3.83	0.05

^∗^ *p* value is from chi-square test for the between-group differences on scanner and gender, and two-sample *t*-test for continuous variables; positive *t* value indicates NC > MCI. ^∗∗^The value is the total distance error in corresponding SN tasks. Note that the higher value for SN distance error indicates a greater total error within a given task. ^∗∗∗^T = Achieva TX; I = Ingenia scanner type.

**Table 2:** Between-group differences in subcortical rs-fMRI measures and volumes; mean (SD).

Region	Measure	Left hemisphere				Right hemisphere			
		NC	MCI	*t*	*p*	NC	MCI	*t*	*p*
Thalamus	ALFF	2.85 ± 0.97	2.41 ± 0.95	0.97	0.34	2.80 ± 0.95	2.37 ± 0.89	1.12	0.27
	fALFF	0.337 ± 0.039	0.323 ± 0.042	0.69	0.50	0.335 ± 0.039	0.323 ± 0.042	1.38	0.18
	ReHo	0.150 ± 0.029	0.135 ± 0.027	2.00	0.05	0.149 ± 0.030	0.134 ± 0.026	2.24	0.03^∗^
	Volume	7.08 ± 1.12	7.08 ± 1.17	0.09	0.93	6.88 ± 0.95	6.86 ± 1.05	0.14	0.89

Caudate	ALFF	2.35 ± 0.84	2.05 ± 0.82	0.51	0.61	2.27 ± 0.81	1.96 ± 0.78	0.49	0.62
	fALFF	0.335 ± 0.039	0.326 ± 0.039	−0.18	0.86	0.336 ± 0.039	0.327 ± 0.040	−0.53	0.60
	ReHo	0.135 ± 0.028	0.135 ± 0.019	0.52	0.60	0.141 ± 0.037	0.139 ± 0.023	0.50	0.62
	Volume	3.05 ± 0.57	3.18 ± 0.39	0.16	0.87	3.35 ± 0.53	3.37 ± 0.38	0.29	0.07
Putamen	ALFF	2.41 ± 0.83	2.12 ± 0.86	−0.22	0.83	2.26 ± 0.85	1.98 ± 0.76	0.52	0.61
	fALFF	0.335 ± 0.038	0.324 ± .039	0.02	0.98	0.335 ± 0.037	0.323 ± 0.040	1.55	0.13
	ReHo	0.134 ± 0.025	0.132 ± .022	0.85	0.40	0.146 ± 0.035	0.134 ± 0.024	1.45	0.16
	Volume	4.40 ± 0.96	4.55 ± 0.73	0.62	0.54	4.36 ± 0.80	4.50 ± 0.71	0.12	0.90

Pallidum	ALFF	2.55 ± 0.87	2.23 ± 0.91	−0.03	0.97	2.27 ± 0.91	2.11 ± 0.82	0.71	0.48
	fALFF	0.328 ± 0.039	0.321 ± 0.041	−0.40	0.69	0.334 ± 0.038	0.321 ± 0.041	1.84	0.07
	ReHo	0.125 ± 0.026	0.117 ± 0.028	0.92	0.36	0.137 ± 0.032	0.116 ± 0.031	2.23	0.04^∗^
	Volume	1.86 ± 0.51	1.82 ± 0.37	−0.03	0.97	1.81 ± 0.46	1.83 ± 0.38	−0.73	0.47

Hippocampus	ALFF	3.22 ± 1.29	2.58 ± 1.03	0.74	0.46	3.00 ± 1.06	2.48 ± 1.00	1.08	0.28
	fALFF	0.328 ± 0.037	0.323 ± 0.043	0.52	0.61	0.338 ± 0.035	0.321 ± 0.044	1.19	0.24
	ReHo	0.131 ± 0.025	0.125 ± 0.026	1.73	0.09	0.135 ± 0.025	0.118 ± 0.026	2.75	0.01^∗^
	Volume	3.14 ± 0.67	3.41 ± 0.54	−1.23	0.23	3.47 ± 0.83	3.57 ± 0.51	−0.18	0.86

Amygdala	ALFF	3.23 ± 1.30	2.58 ± 1.00	0.31	0.76	2.91 ± 0.99	2.47 ± 0.93	0.92	0.38
	fALFF	0.335 ± 0.036	0.324 ± 0.042	0.11	0.92	0.339 ± 0.033	0.325 ± 0.039	0.84	0.41
	ReHo	0.126 ± 0.024	0.121 ± 0.029	1.36	0.18	0.132 ± 0.029	0.112 ± 0.029	2.98	0.01^∗^
	Volume	1.05 ± 0.38	1.02 ± 0.29	−0.72	0.47	1.15 ± 0.26	1.16 ± 0.23	−0.31	0.76

Accumbens	ALFF	2.61 ± 0.88	2.27 ± 0.87	0.09	0.93	2.60 ± 0.96	2.23 ± 0.85	0.63	0.53
	fALFF	0.336 ± 0.038	0.325 ± 0.040	−0.20	0.84	0.338 ± 0.044	0.323 ± 0.043	0.20	0.84
	ReHo	0.124 ± 0.034	0.118 ± 0.024	1.17	0.25	0.129 ± 0.033	0.118 ± 0.030	0.75	0.46
	Volume	0.42 ± 0.17	0.42 ± 0.14	0.87	0.39	0.27 ± 0.14	0.31 ± 0.10	−0.12	0.90

The subcortical volumes are described in milliliter (ml). The rs-fMRI measures are in natural units. *p* value is from two sample *t*-tests adjusted for age, gender, scanner type, and years of education (and intracranial volume for all subcortical volumes). Positive *t* value indicates NC > MCI. ^∗^*p* < 0.05.

**Table 3:** Correlations between ReHo and spatial navigation accuracy in the right thalamus, hippocampus, pallidum, and amygdala.

Navigation accuracy		R thalamus		R pallidum		R hippo		R amygdala	
		*r*/*F*	*p*	*r*/*F*	*p*	*r*/*F*	*p*	*r*/*F*	*p*
Allo-Ego	Within MCI	0.39	0.11	0.43	0.07	0.29	0.24	0.27	0.28
	Within NC	−0.35	0.17	−0.26	0.31	−0.33	0.19	−0.35	0.17
	Interaction	3.04	0.09	1.51	0.23	3.22	0.08	2.49	0.12

Ego	Within MCI	−0.12	0.63	0.40	0.10	0.21	0.40	0.17	0.50
	Within NC	−0.03	0.90	−0.04	0.86	−0.17	0.50	−0.23	0.38
	Interaction	0.21	0.64	1.00	0.32	1.09	0.30	1.71	0.20

Allo	Within MCI	0.21	0.41	0.32	0.20	0.18	0.46	0.19	0.44
	Within NC	−0.49	0.05^∗^	−0.50	0.04^∗^	−0.45	0.07	−0.38	0.13
	Interaction	5.95	0.02^∗^	8.97	0.01^∗^	4.44	0.04^∗^	2.82	0.10

For “within MCI” and “within NC” rows, the *r*/*F* columns, the “*r*” (correlation coefficients) represents the correlation between ReHo and respective SN total distance error. For “interaction” rows, the *F* value represents the effect of clinical diagnosis (MCI or NC) on this correlation. ^∗^*p* < 0.05.
